# Environmental effects on molecular and phenotypic variation in populations of *Eruca sativa* across a steep climatic gradient

**DOI:** 10.1002/ece3.646

**Published:** 2013-06-24

**Authors:** Erik Westberg, Shachar Ohali, Anatoly Shevelevich, Pinchas Fine, Oz Barazani

**Affiliations:** 1 Institute of Plant Sciences, Israel Plant Gene Bank, Agricultural Research Organization50250, Bet Dagan, Israel; 2 Institut für Spezielle Botanik und Botanischer Garten, Johannes Gutenberg-Universität MainzD-55099, Mainz, Germany; 3 The Robert H. Smith Institute of Plant Sciences and Genetics in Agriculture, The Robert H. Smith Faculty of Agriculture, Food and Environment, The Hebrew University of Jerusalem76100, Rehovot, Israel; 4 Department of Soil Chemistry, Plant Nutrition and Microbiology, Institute of Soil, Water and Environmental Sciences, Agricultural Research Organization50250, Bet Dagan, Israel

**Keywords:** Environmental adaptation, *Eruca sativa*, genetic diversity, outlier loci, phenotypic variation

## Abstract

In Israel *Eruca sativa* has a geographically narrow distribution across a steep climatic gradient that ranges from mesic Mediterranean to hot desert environments. These conditions offer an opportunity to study the influence of the environment on intraspecific genetic variation. For this, we combined an analysis of neutral genetic markers with a phenotypic evaluation in common-garden experiments, and environmental characterization of populations that included climatic and edaphic parameters, as well as geographic distribution. A Bayesian clustering of individuals from nine representative populations based on amplified fragment length polymorphism (AFLP) divided the populations into a southern and a northern geographic cluster, with one admixed population at the geographic border between them. Linear mixed models, with cluster added as a grouping factor, revealed no clear effects of environment or geography on genetic distances, but this may be due to a strong association of geography and environment with genetic clusters. However, environmental factors accounted for part of the phenotypic variation observed in the common-garden experiments. In addition, candidate loci for selection were identified by association with environmental parameters and by two outlier methods. One locus, identified by all three methods, also showed an association with trichome density and herbivore damage, in net-house and field experiments, respectively. Accordingly, we propose that because trichomes are directly linked to defense against both herbivores and excess radiation, they could potentially be related to adaptive variation in these populations. These results demonstrate the value of combining environmental and phenotypic data with a detailed genetic survey when studying adaptation in plant populations.

This article describes the use of several types of data to estimate the influence of the environment on intraspecific genetic variation in populations originating from a steep climatic gradient. In addition to molecular marker data, we made use of phenotypic evaluation from common garden experiments, and a broad GIS based environmental data with edaphic information gathered in the field. This study, among others, lead to the identification of an outlier locus with an association to trichome formation and herbivore defense, and its ecological adaptive value is discussed.

## Introduction

The East Mediterranean region is considered a biodiversity hotspot, being on the edge of the southern and northern distribution ranges of temperate and desert species, respectively, as well as the distribution center of many others (Roll et al. 53). The region's location at the border of three main phytogeographical regions – Mediterranean, arid Saharo-Arabian, and steppic Irano-Turanian – the wide range of habitats available, and the steep gradient in climatic conditions have contributed to its rich floristic biodiversity (Danin and Plitmann 15). Accordingly, it has been postulated that the habitat variations in the East-Mediterranean region provide opportunities for genetic substructuring and the establishment of localized genetic diversity and adaptations in plant populations (Nevo 42). As such, extreme environments enable evolutionary changes that can lead to local adaptation and speciation but also to extinction (Nevo 43), and several studies demonstrated the effects of selective factors, for example, stressful abiotic environments, on phenological and morphological divergence (Aronson et al. 2, 3; Volis et al. 68; Petru et al. 51; Liancourt and Tielborger 36). For local adaptation to arise, selection must be strong enough to overcome the homogenizing effects of gene flow (Slatkin 58). Moreover, adaption to a specific habitat can be influenced by the spatial heterogeneity of the environment, for example, climatic and edaphic conditions, which may result in ecotypical or clinal patterns of phenotypic differentiation (Turesson 62; Silberbush et al. 57; delaVega 64).

It is well known that several factors influence the genetic structure and levels of genetic variation among plant populations. Past reviews have shown that dispersal biology, mating system, life form, and geographic range all affect the distribution of genetic variation within and among populations (Hamrick and Godt 24, 25; Nybom and Bartish 48). Other factors that influence genetic variation include: the demographic history of a species (population bottlenecks and expansions, recent establishment or long persistence of populations), population size, and current gene flow. Whereas such factors can affect the whole genome, selection, in contrast, acts mainly on individual traits. Therefore, neutral variation does not necessarily reflect the phenotypic variation (Reed and Frankham 52; Vazquez-Garciduenas et al. 63; Hughes et al. 29), and selection is often not considered amenable for study with neutral markers. However, recent attention has been given to questions about the influence of local conditions on genetic variation, and it has been shown that local adaptation can influence neutral variation by giving rise to reproductive barriers (Nosil et al. 46).

A considerable amount of research has been focused on the characterization of genetic diversity in the Near East, including the associations between phenotypic variation and environmental factors with local adaptations (Volis et al. 70; Petru et al. 51; Liancourt and Tielborger 36). For example, along the aridity gradient in Israel, differences among populations have been found in seed morphology and germination (Aronson et al. 4; Volis 67; Barazani et al. 6). Switches to early reproduction were also associated with annuals in drier environments in the Near East (Aronson et al. 3; Nevo et al. 44) and neutral diversity has also been examined in various plant species and has often been found to be associated with either climatic or geographical conditions (Comes and Abbott 10; Arafeh et al. 1; Volis et al. 69; Nahum et al. 40; Peleg et al. 50; Samocha et al. 56). Here, we present a survey of genetic diversity and phenotypic traits in populations of *Eruca sativa* Mill. (syn *E*. *vesicaria* subsp. *sativa*, Brassicaceae) and their association with geographic and environmental conditions in search for adaptive variation.

**Figure 1:**
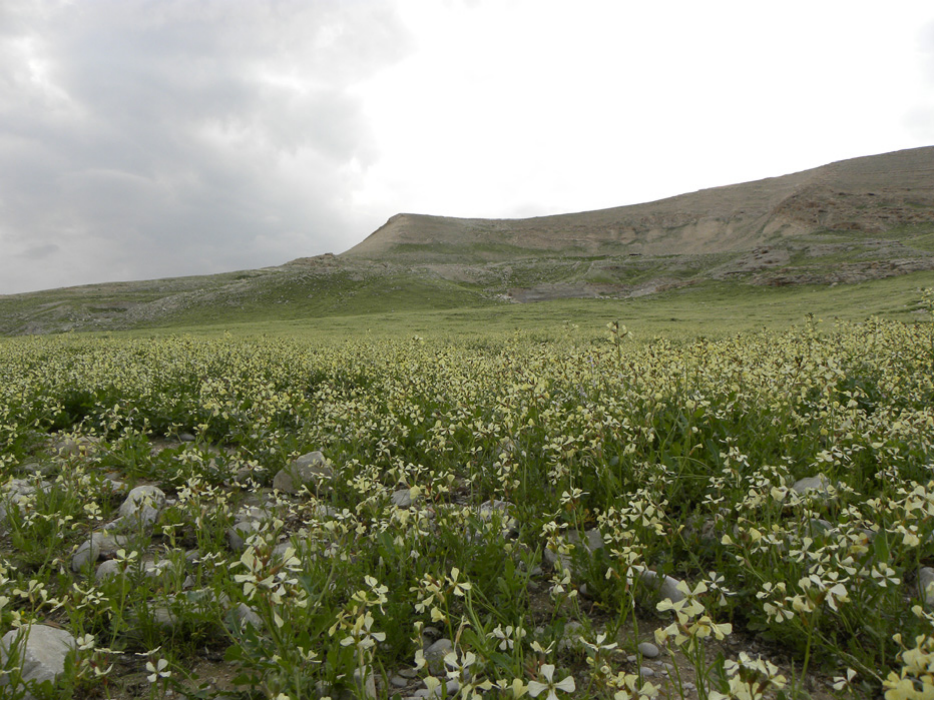
A population of *Eruca sativa* in the Jordan Valley desert habitat.

**Figure 2:**
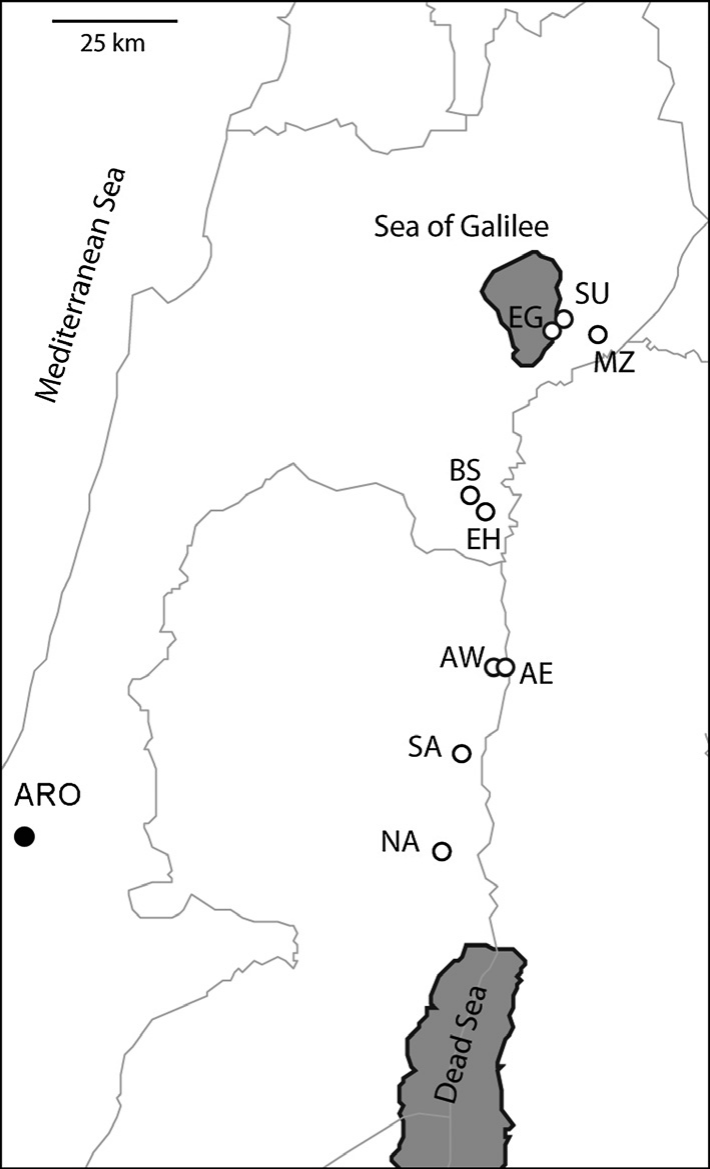
Locations of *Eruca sativa* populations and of Agricultural Research Organization where phenotypic evaluation was performed in common-garden and net-house experiments.

*Eruca sativa* is an insect-pollinated self-incompatible winter annual species with pale cream to yellow flowers. Its occurrence in Israel is at the easternmost Mediterranean periphery, where populations are distributed in a narrow geographical range from the southern Golan Heights in the north to the Jordan Valley in the south (Fig. 1), across a steep climatic gradient, from mesic Mediterranean conditions in the north to the arid-hot desert conditions in the south (Table 1; Fig. 2). In the latter habitats, *E. sativa* is the dominant species, with about 20–50% vegetation cover, whereas, in contrast, the more favorable conditions in the northern habitats support dense annual-plant communities in which *E. sativa* forms only a minor component of the total vegetation cover. Furthermore, populations of *E. sativa* in the southern arid habitats showed a much smaller morph (appendix Excel file) and also showed earlier flowering than those in populations of more mesic habitats (O. Barazani, pers. obs.). We therefore set out to investigate whether patterns of molecular and phenotypic variation in *E. sativa* populations reflect adaptations to the strong environmental gradient. To explore this possibility, we used amplified fragment length polymorphism (AFLP) markers and characterized phenotypic traits and environmental conditions in populations of *E. sativa*, in order to address the following questions: (1) what is the role of the environmental and geographical conditions in structuring neutral genetic variation? (2) do these factors affect genotypic and phenotypic variation? (3) do candidate loci for selection associate with local environmental conditions?

**Table 1:** Populations of *Eruca sativa* ordered from north to south, genetic diversity within populations and the climatic conditions at the natural sites with the two first principal components of PCA analysis (cf. Fig. S2)

Population		Genetic diversity^1^					Coordinates							Altitude (m)	Avg. temp. (°C)^2^	Avg. annual rainfall (mm)	PC1	PC2
*n*	P	uHe	Latitude	Longitude														
Susita	SU	14	61.14	0.196	32°46′39″	35°39′29″	40	11.8	430	−3.660	−1.804
Ein Gev	EG	15	60.26	0.199	32°46′09″	35°38′36″	−167	12.4	390	−1.859	−1.714
Meizar	MZ	15	65.94	0.205	32°45′38″	35°41′16″	175	10.8	516	−5.149	1.701
Bet Shean	BS	15	62.88	0.200	32°30′04″	35°30′38″	−166	12.9	330	−0.521	1.437
Ein ha'Naziv	EH	15	57.21	0.192	32°28′01″	35°30′39″	−188	13.0	310	−0.041	1.042
Argaman (west)	AW	14	60.26	0.202	32°13′19″	35°33′01″	−273	13.6	198	2.816	0.673
Argaman (east)	AE	15	62.88	0.199	32°13′24″	35°33′37″	−330	13.8	195	2.939	−0.903
Sartaba	SA	15	63.32	0.200	32°04′49″	35°29′46″	−278	13.6	200	3.172	1.744
Na'ama	NA	13	60.70	0.209	31°55′31″	35°27′52″	−235	13.6	155	2.303	−2.176

1 P, percentage of polymorphic loci; uHe, unbiased expected heterozygosity.

2 Average daily temperature during the growing season (January).

## Methods

### Plant material

Nine populations of *E. sativa* were mapped along the plant's geographical distribution range in Israel: along the Jordan Valley, the east coast of the Sea of Galilee, and the southern slopes of the Golan Heights (Table 1, Fig. 2). Leaf samples were collected from at least 15 individual plants at each site, with a separation of at least 4 m between plants. At the end of the growth season, seeds were collected from at least 30 plants, pooled together and stored at room temperature. The leaf samples were used for DNA extraction; the seeds were used for phenotypic evaluation in experiments under field and net-house conditions.

### DNA extraction and AFLP analysis

DNA was extracted with the DNeasy plant mini kit (QIAGEN, Hilden, Germany). The restriction, ligation, and preselective PCR of the AFLP procedure followed Vos et al. (71) with the modification of Kropf et al. (32). Approximately 150 ng of DNA was simultaneously restricted and ligated to adaptors (EcoRI, 5′-CTCGTAGACTGCGTACC-3′/5′-A ATTGGTACGCAGTC-3′; MseI, 5′-GACGATGAGTCCTGAG-3′/5′-TACTCAGGACTCT-3′) at 23°C for 14 h. Preselective amplification was performed with the primers E01 (5′-GACTGCGTACCAATTCA-3′) and M02 (5′-GATGAGTCCTGAGTAAC-3′). The selective amplifications followed Trybush et al. (61). For these amplifications three labeled EcoRI-primers and one unlabeled MseI-primer were used simultaneously for multiplex PCR (QIAGEN). The primers E37 (E01 + CG), E39 (E01 + GA), and E45 (E01 + TG) were used in combination with M54 (M02 + CT) and M55 (M02 + GA) to provide six different combinations. The AFLP products were separated on an ABI 3100 automated sequencer (Applied Biosystems, ABI, Weiterstadt, Germany) as a multiplex of three primer combinations labeled with fluorescent dyes (6-FAM, NED, and HEX; Applied Biosystems, ABI) together with an internal size standard labeled with ROX (ROX 500; Applied Biosystems, ABI).

Electropherograms were scored automatically with Genmarker 1.75 (SoftGenetics, State College, PA) and corrected manually. Extra care was taken to avoid scoring fragments twice that were labeled with two different dyes, and ambiguities were recorded as missing data. Genotyping error rates were estimated with 15 replicated samples by dividing the number of mismatches between replicates by the total number of phenotypic comparisons (Bonin et al. 9).

### Phenotypic evaluation

Pooled seed samples of each population were germinated on moistened filter paper, and randomly selected seedlings from each population were transplanted and grown in the experimental field site at the Agricultural Research Organization (ARO), Bet Dagan, Israel (32° 46′ 39″ N, 35° 39′ 28″ E; ca. 50 m above sea level), outside the geographical study area (Fig. 2). An additional group of plants was transplanted to 1-L pots containing potting medium (Shaham, Israel) that comprised 50% peat, 30% tuff, and 20% perlite, and grown in an insect-free net-house located at ARO. In the field experiment (herein common-garden), plants were rain irrigated during the rainy season (December–March), during which rainfall was about 350 mm. In the net-house, plants were irrigated with a computer-controlled trickle irrigation system that supplied water at 2 L/h for 1 min twice daily. Altogether the common-garden experiment included 20–24 plants and the net-house experiment 16 plants from each of the various populations. Plants were grown in a randomized block design, and were phenotypically evaluated from the vegetative stage to flowering.

Phenotypic evaluation in the common-garden experiment included recording of the day of bolting (when the first elongated flower stem reached 1 cm in height), the first day of flowering, and stalk height at the end of the growth stage. Qualitative estimation of trichome density on buds and stems was estimated on a scale of 0 – glabrous to 5 – highly dense. Herbivore damage in the common-garden experiment was estimated at the rosette stage on a scale of nil damage (0) to severely damaged (5). Qualitative evaluation of intensity of yellow pigmentation in the flowers was evaluated in a scale of pale cream (1) to bright yellow (4). Examples of qualitative traits are given in Fig. S1. In the net-house, the number of rosette leaves on each plant was counted, and the rosette diameter was measured on the day the plants started their elongation stage. Stalk lengths were measured every 3 days, and the days of bolting and of the first flowering were noted. Trichome density on buds and stems was estimated qualitatively, and the number of inflorescences on each plant was counted.

The phenotypic evaluation was performed by one person. All plants used in the phenotypic evaluations were raised from seed pools collected in nature, and thus the possibility of maternal effects cannot be excluded.

### Climatic and edaphic conditions

Climatic environmental data at the sites where populations were sampled were obtained from the Israel Biodiversity and Information System (the Hebrew University of Jerusalem, Israel). These data included annual temperatures, precipitation, altitude, and slope (Tables 1 and S1).

For characterization of the edaphic conditions, we collected soil samples from the upper 10-cm soil layer in at least eight locations at each population site. Samples were taken with a metal auger (5.5 cm in diameter) with a separation of at least 4 m between samples. The soil samples were dried at room temperature, crushed gently, and sieved to pass a 2-mm screen, in order to qualitatively separate and weigh the stones and the <2-mm fractions. The latter were analyzed as follows for edaphic properties: (1) pH and electrical conductivity (EC), as an expression of salinity, were measured in the supernatant, following shaking 15-g soil samples in 30 mL of demineralized water on a reciprocal shaker for 1 h and centrifuging at 3100 g; (2) Carbonates (as CaCO_3_) were assessed by measuring the amount of CO_2_ gas evolved from a 100-mg soil sample in a constant-pressure calcimeter (Machette 37); (3) Hygroscopic water content (HMC) was calculated from the weight loss from air dried 10-g soil samples after heating for 16–24 h in an oven at a temperature of 105°C. The resulting data were used to calculate the soil-specific surface area (SSA) using the regression coefficient provided by Banin and Amiel (5): HMC (in % w/w) = 0.025 × SSA + 0.488 (R^2^ = 0.949) (Banin and Amiel 5); (4) Loss on ignition (LOI) was calculated from the change in weight of soil samples that had been oven dried at 105°C for 24 h, after heating for further 8 h at 500°C. The analyses were performed in duplicate on each of the collected soil samples.

### Genetic data analysis

Genetic diversity was calculated using GenAlEx v.6.4 (Peakall and Smouse 49) as the percentage of polymorphic loci (P), and the unbiased expected heterozygosity (He) assuming Hardy–Weinberg equilibrium. Spatial genetic structure was analyzed with a Bayesian, individual-based clustering method using BAPS 5.4 (Corander and Marttinen 11; Corander et al. 12). The analysis was run 10 times each for *K* = 2–10, where *K* is the assumed maximum number of clusters in the data. An admixture analysis of individuals from one population was subsequently carried out using BAPS 5.4 to estimate the genetic ancestry of individuals. This analysis was done using predefined clusters based on the initial mixture results. Significance of admixture coefficients was estimated by comparing them to coefficients from 200 individuals simulated from source populations whose allele frequencies matched those of the populations inferred in the preceding cluster analysis (Corander and Marttinen 11).

A clustering of populations was done with the unweighted pair group method with arithmetic mean (UPGMA) method based on Nei's genetic distances (Nei 41), using the TFPGA software (Miller 38). Node support was estimated by bootstrapping the data (*N* = 1000). To quantify differentiation, analyses of molecular variance based on band frequencies (Excoffier et al. 16) was performed using Arlequin 3.0 (Excoffier et al. 17). Genetic variation was partitioned among populations, among individuals, and among BAPS clusters.

To test for associations between geographic and environmental distances and genetic variation, linear mixed models (LMMs) were used. In keeping with regression methods commonly used in IBD (isolation by distance) analysis, genetic distances were measured as *F*_ST_/(1 − *F*_ST_) and geographic distances were ln transformed (Rousset 54). The climatic data and the site means for the edaphic data were subjected to a principle components analysis based on the correlation matrix principal component analysis (PCA) using the software PAST (Hammer et al. 23) and the Euclidean distance between sites along the first four axes was used as the environmental distance. Because geographical genetic substructure can have a substantial effect on IBD patterns, and on any other pattern that correlates with distance (Husband and Barrett 30; Cushman et al. 14; Cushman and Landguth 13) and yield spurious correlations, we controlled for population substructure by adding it to the LMM as a grouping variable, where pairwise distances within clusters, between clusters and between the admixed population and the other clusters were assigned to different categories. The environmental and geographical distances were entered as fixed effects. Model analysis and significance testing of the fixed effects were performed with the lme4 package (Bates et al. 7) in R (R Core Team 100). Significance was estimated by fitting a reduced model (null model), that is, one not containing the variables of interest, to the genetic distance data and then simulating response data based on this reduced model. Both the reduced and the full model were fitted to the simulated data, and the difference between the two models in the log-likelihood was calculated. Response data were simulated 1000 times to generate a bootstrap distribution for the null hypothesis that the reduced model was the true one. The observed difference in likelihood of the original data was then compared with this distribution. *R*^2^-values for the models were estimated from the correlation between the observed and fitted values. Multiple regressions on the distance matrices, without accounting for clustering results, were also performed for comparison.

### Phenotypic data analysis

The effects of the environmental conditions and geographical distances on phenotypic variation were analyzed with multiple regressions. Environmental distances were based on the four first axes of a PCA as above. The same was done for the population means obtained from the phenotypic evaluation and used as the phenotypic distance. Significance of the regression coefficients and correlation coefficients were estimated with the multiple regression on distance matrices (MRM) function in the R-library ecodist (Goslee and Urban 21). To test whether both geographic and environmental distances added significantly to the model, a stepwise forward selection was performed using Permute!3.4 with *P* to enter = 0.05 (Legendre et al. 35). This method adds explanatory variables to the regression model and tests them one at a time, beginning with the one with the most significant correlation value (*R*^2^). Significance testing was done with permutation tests. The regression models considered were: (1) Phenotypic distance (net-house) = (geographic distance + environmental distance) and (2) phenotypic distance (common-garden experiment) = (geographic distance + environmental distance). The common-garden and the net-house data were tested separately as data for one locality of each experiment were missing.

In cases where environmental data were found to have a significant relationship with the genetic or phenotypic distance, the relative importance of the climatic and edaphic subsets of the environmental data was investigated with partial Mantel tests (Legendre and Legendre 34). For this, the environmental data subsets were separately subjected to PCAs, and Euclidean distances along the first four axes were used to calculate partial matrix correlations, while controlling for geographic distance and genetic cluster.

### Candidate loci

To identify candidate loci, potentially influenced by selection, three different methods were used. First, the *F*_ST_ outlier method, as implemented in Dfdist software (a modification of Ddist2 for dominant data) (Beaumont and Nichols 8) was used to detect AFLP markers that showed signs of divergent selection. Global population *F*_ST_ values were calculated locus-by-locus and compared with a neutral expectation derived from simulations. Markers that were significantly more differentiated than expected were regarded as candidates for being influenced by divergent selection. The target *F*_ST_ for the simulations (*N* = 50,000) was estimated from the AFLP data using the untrimmed mean, and theta was set to 0.018. Varying theta between 0.009 and 0.09 did not affect the results significantly.

Second, an alternative outlier method implemented in the Bayescan 2.1 program was used (Foll and Gaggiotti 19). This Bayesian method decomposes locus-population *F*_ST_ values into a population-specific and a locus-specific component. Departure from neutrality is inferred when the locus-specific component is necessary to explain the pattern of diversity seen in the data. This is done by comparing posterior probabilities of a model with the locus-specific component with those of a model without it. Rather than using *P*-values, the authors of the program recommend the use of *q*-values and outlier was regarded significant at a threshold *q*-value of 5%. A *q*-value for a locus is the expected proportion of false positives inferred when the locus is regarded as significant.

Finally, to test for associations between the environment and AFLP markers, we used the spatial analysis method (SAM) (Joost et al. 31). This program uses multiple univariate logistic regressions to test for associations between marker frequencies and environmental variables. Both a Wald-test and a *G*-test are calculated and an association is considered significant only if both tests reject the null hypothesis at the chosen Bonferroni-corrected significance level. Environmental variables were not entered individually because there were several relatively strong correlations among them. Instead, the coordinates for populations along the first two axes of the PCA, which corresponded strongly to climate and edaphic conditions, respectively, were used. This yielded a total of 372 models and a Bonferroni-corrected threshold of 2.69 × 10^−5^, corresponding to a *P*-value of 0.01.

## Results

### Environmental variation among sites

In the northern sites, populations of *E. sativa* can be found below sea level, along the east coast of the Sea of Galilee (population EG, 167 m below sea level) and at higher altitudes on the southern slopes of the Golan heights (populations SU and MZ, 40 and 175 m above sea level, respectively), all with an average annual rainfall >400 mm. Along the Jordan Valley, populations of *E. sativa* grow at low altitudes (>165 m below sea level) and in an aridity gradient that ranges from semiarid (BS and EH, about 300 mm average annual rainfall) to arid (AW, AE, SA and NA, with ≤200 mm/year) conditions (Table 1).

Soil characteristics of the southern Jordan Valley differ from these of the northern (MZ, EG, and SU) sites. In general, the southern sites are more saline (higher EC values), but contain smaller stone fractions (stoniness values) (Table S1). The soil LOI did not show a clear pattern, with values being lower in the north and south (Table S1).

The contents of carbonates (as CaCO_3_) in the populations on the slopes facing the Sea of Galilee (EG, SU) were markedly lower than those of other populations, but no differences were observed between the reactions (PH) of the soils (Table S1). The assumed SSA values in soils originating from populations EG and SU (53 and 65 m^2^/g, respectively) were similar to that of EH, AE, and NA, but lower than those in the other Jordan Valley soils (>100 m^2^/g), indicating a higher clay fraction in the latter soils.

Principal component analysis of the correlation matrix of the environmental variables divided the populations on the basis of topography, and climatic and edaphic conditions (Fig. S2). The first two axes of the PCA accounted for 75.6% of the variation, mostly affecting the differentiation between the southern arid sites of the Jordan valley (SA, AW, AE, and NA) and the semiarid sites (BS and EH), and between the latter two and the Mediterranean mesic habitats (EG and SU) in the northern distribution range of the species (Fig. S2). The first principal component was dominated by climatic conditions, especially temperature and rainfall, and the second component was correlated mostly with variations in soil parameters, such as soil surface area, calcium carbonate content, LOI, and stoniness, but also with topographic parameters, for example, slope and aspect (Fig. S2).

### Phenotypic variation among populations

The phenotypic characteristics varied significantly among populations for all measured traits; the data are supplied as an appendix Excel file. In general, sites with low vegetation cover, for example, NA, SA, and SU, showed earlier bolting and flowering than other sites, in both the net-house and the common-garden experiments, but no trend was found in the heights of the plants. Trichome density on buds and stems was lower in populations from northern sites than in those from the southern sites in the net-house but these differences were diminished in the common-garden experiment. In addition, the southern populations showed a tendency to be less damaged by herbivory than the central or northern ones. However, many of the traits varied unpredictably among the sites, or showed partially contrasting patterns between the common-garden and the net-house experiments.

A principal components analysis of the phenotypic data from the net-house and the common-garden experiments is shown in Fig. S3. In the net-house experiment, the first two axes accounted, respectively, for 44% and 28% of the variation (Fig. S3A): the first axis correlated mainly with flowering and bolting dates, stalk height, and the number of inflorescences; the second was dominated by stem and bud trichomes and rosette diameter (Fig. S3A). For the common-garden data, the first two axes accounted for 35% and 30% of the variation (Fig. S3B): the first axis corresponded mainly to flowering and bolting dates and early herbivore damage; the second to late herbivore damage, stalk height, and trichome density (Fig. S3B).

### Genetic diversity among populations

The genetic analysis covered 131 individuals, originating from nine *E. sativa* populations. The AFLP analysis, with six different primer combinations, yielded 229 loci that could be reliably scored, of which 81% were polymorphic at the 99% level. The mismatch error rate was low, at 0.78%. Genetic diversity within populations, expressed as unbiased expected heterozygosity (He), was similar in all the sampled populations (Table 1). The same general result was obtained when diversity was measured as percentage of polymorphic loci (Table 1).

**Figure 3:**
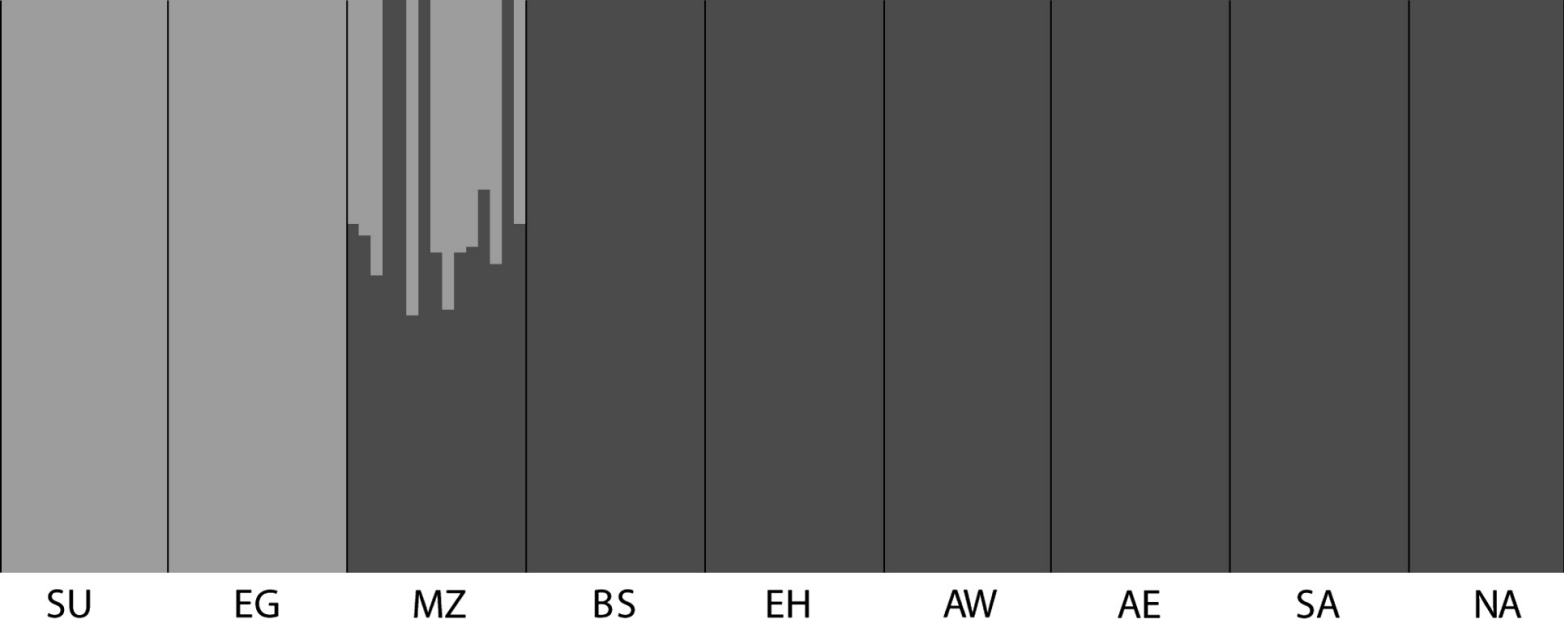
Inferred clusters of individual genotypes and admixture results for MZ, as calculated with BAPS software, for populations of *Eruca sativa*. Each vertical bar represents one individual genotype. Individuals with two colors have admixed genotypes from the two main southern and northern genotype clusters. The admixture analysis was done with the eight unmixed populations used as ancestral gene pools.

The mixture analysis (Fig. 3) and the UPGMA dendrogram (Fig. S4) divided the nine populations into two main phylogeographic groups (posterior probability of two clusters = 1), representing a northern (SU, EG) and a southern (MZ, BS, EH, AW, AE, SA, NA) geographic group. The two populations of the northern cluster were situated on the southern Golan Heights and on the east coast of the Sea of Galilee, respectively; the southern cluster was predominantly in the semi-arid/arid area of the Jordan Valley, with the exception of MZ, which has a Mediterranean climate, much like that of the two populations of the northern cluster (Table 1). Most of the above populations contained individuals belonging to a single cluster, but that of MZ was found to be mixed. An admixture analysis of the MZ population suggested that the individuals from MZ either originated from the southern cluster or were of mixed genetic ancestry (*P* ≤ 0.01 for all admixed individuals; Fig. 3). A nonhierarchical analysis of molecular variance (AMOVA) partitioned 9.48% of the variation among populations (*P* < 0.001), a hierarchical AMOVA partitioned 8.26% of the variation between the two genetic clusters (*P* = 0.030), and 5.8% was partitioned among populations within each of the two groups (*P* < 0.001).

### Influence of environment and geographic conditions on genotypic and phenotypic variation

Multiple regressions revealed that both the environmental and geographic distances showed significant positive relationships with genetic distances. The environmental distances accounted for more of the variance than geographic distances (*R*^2^ = 0.33; *P* = 0.003; and *R*^2^ = 0.19, *P* = 0.039, respectively), and the variance explained when adding both variables was only a fraction higher (*R*^2^ = 0.340). A stepwise regression showed that each of the variables significantly improved the model.

In contrast, the LMM showed that the genetic cluster had a large impact on the pattern of genetic diversity and, by itself, could almost entirely account for the positive correlations seen with geography and environment. Thus, adding environment and geography to a model containing only cluster raised the explained variance only very slightly, from *R*^2^ = 0.884 to *R*^2^   0.886 (Table 2). The simulations showed that neither adding environment and geography alone, nor adding them both improved the model significantly (Table 2).

**Table 2:** Significance test and *R*^2^ values for environmental and linearized geographic distances with data grouped by genetic cluster

Reduced model (explanatory variables)	Full model (additional variables)	*R* ^2^	*P*
	Subgroup	0.884	
Cluster	Geographic distance	0.886	0.50
Cluster + geographic distance	Environmental distance	0.886	0.81

Phenotypic data from the net-house and the common-garden experiments showed differing patterns. The PCA distances based on data from the common-garden experiment were positively related to both environmental and geographic distances. However, a stepwise regression selected only environmental distance, whereas geographic distance did not improve the model significantly (Table 3). In contrast, comparison between PCA distances obtained from the net-house experiment, on the one hand, and environmental and geographical distances, on the other hand did not yield significant regression coefficients (Table 3). Partial Mantel tests controlling for geographic distance and genetic cluster showed that more phenotypic variation was accounted for by edaphic factors than by climatic factors in the common-garden experiment (*r*_edaphic_ = 0.48, *P* = 0.020, *r*_climatic_ = 0.100, *P* = 0.730).

**Table 3:** Intercepts, regression coefficients, and *R*^2^ values of phenotypic distances of data from net-house and common-garden experiments as dependent variables with environmental distances and linearized geographical distances as the explanatory variables

Dependent variable	Intercept	Environmental distance	ln(distance)	Pearson *R*^2^
Net-house	1.699	0.054^ns^	−0.058^ns^	0.025
	1.600	0.034		0.018
	1.731		0.013	0.001
Common-garden	1.800	0.201**	0.168^ns^	0.280*
	2.063	0.269		0.257**
	2.180		0.378	0.180*

Asterisks indicates significant levels at ^*^P <* *0.05, and ^*^^*^0.01; nonsignificant (ns) results are indicated by superscript letters. Significance tests for regression coefficients in the full models were estimated by forward stepwise addition using Permute!3.5 (Legendre et al. 35), and *R*^2^ values were tested using the MRM script in the Ecodist R-library (Goslee and Urban 21).

### Detection of candidate loci

The Dfdist analysis was applied to 186 polymorphic loci. The mean *F*_ST_ over all loci was 0.067. Two loci had *F*_ST_ values higher than the 99% confidence interval. Repeating the analysis after removal of these two loci revealed one additional candidate locus and, therefore, three loci were potentially affected by divergent selection (Table 4). No outliers with lower *F*_ST_ than expected were detected by the analysis. The number of neutral markers with a *P*-value below 0.5 (*N* = 88) was similar to the number of markers with higher *P*-values (*N* = 95), confirming that the simulation model was appropriate (Dfdist manual). Analysis with Bayescan yielded four candidate loci significant at the *q*-value threshold of 5%, all of which showed signs of diversifying selection. Two of these were also found in the Dfdist analysis and thus a total of five outliers were detected by the analyses (Table 4); the mean *F*_ST_ of these outliers were 0.24.

**Table 4:** Nine candidate loci identified by the analyses with Dfdist, Bayescan, and SAM. Fragment names consist of the number of the M-primer, the two last selective bases of the E-primer, and the fragment length

Fragment	Bayescan *q*-value	Dfdist *P*-value	SAM adjusted *P*-value				
			Axis1	Axis2
55GA88	0.803	0.077	0.000*	–
55GA170	0.067	0.001*	–	0.002*
55GA254	0.024*	0.002*	1.000	–
55TG101	0.493	0.033	–	0.001*
55TG138	0.112	0.017	0.009*	–
55CG413	0.576	0.054	0.002*	–
54GA254	0.014*	0.000*	0.000*	–
54TG145	0.001*	0.016	0.180	–
54CG213	0.045*	0.047	1.000	–

* *P*-values below 0.01 (Dfdist, SAM) and *q*-values below 0.05 (Bayescan).

The same data set was analyzed with SAM to detect associations with environmental variables. Four markers were significantly associated with the first, climate-dominated axis, and two markers with the second axis, corresponding to edaphic and topographic factors (Table 4).

## Discussion

This study provides a broad survey of genetic variation along the distribution range of *E. sativa* in Israel. There is substantial variation in environmental conditions along the narrow local geographic distribution range of *E. sativa –* variation that encompasses both climatic conditions (i.e., temperature and rainfall) and edaphic conditions (i.e., conductivity, CaCO_3_ content, and SSA), as well as vegetation. Despite this, genetic diversity and structure was not unambigously associated with environment or geography, instead it was best explained by genetic cluster. Also, the finding of a genetic break that divided the investigated populations into two clusters did not coincide with the most striking differences in morphology observed in nature. Indeed, differences in phenotype largely disappeared in the common-garden experiments, which showed that this variation was almost entirely due to plasticity. Nevertheless, regression analysis showed that part of the phenotypic variation measured under field conditions could be attributed to the environment. In addition, several candidate loci for selection were detected by three different methods, suggesting a role for adaption to environmental conditions.

### Genetic diversity

The present results show a moderate but clear genetic break that subdivides the nine studied populations within the narrow distribution range into a southern and a northern cluster (Figs. 3, S4). Although the existence of two genetic clusters could be attributed to local adaptation to differing climatic conditions, or to the apparent geographical distribution gap that separates the southern from the northern population (Fig. 2), the finding of distinct genetic groups in phylogeographic surveys is usually interpreted in terms of historical demography. Thus, the present two groups could be plausibly considered as reflecting the existence of Irano-Turanian (southern group) and Mediterranean (northern group) elements in the area (Danin and Plitmann 15). The modern distribution range in the Jordan Valley and southern Golan Heights could represent a contact zone between former allopatric groups. A broader phylogeographic study of both introduced and natural populations in the eastern Mediterranean and the Near East is needed to distinguish these hypotheses.

Because the two clusters were located in a north-south pattern, testing for effects of geographic distance without correcting for this spatial pattern might yield spurious regression coefficients. The same applies to environmental factors if they are correlated with the geographic hierarchical structure. This was exemplified by the significant effects of the geographic and environmental distances on genetic distances in multiple regressions, which do not control for hierarchical structure. Cushman and Landguth (13) suggested using causal modeling with partial Mantel tests to identify the process responsible for genetic patterns, and this approach identified cluster as the causal process (data not shown). Conversely, if true effects of geography were correlated with the geographic clusters, correcting for hierarchical structure might remove a large part of these effects (Mulley et al. 39). Analyses subsequent to the partial Mantel tests, however, would have to be restricted to populations belonging to the same cluster, which would lead to a lack of power, because of the reduction of the number of data points. Instead, we here analyzed the full distance matrices with LMM, with substructure added as a grouping variable, and used simulated response data of a null model to estimate significance. An advantage of this approach is that the contribution of all parameters could potentially be estimated by using the whole data set, in case they had independent effects as well as their possible interactions, for example, (Lee and Mitchell-Olds 33). In the end, this approach identified cluster as the major influence on marker variation, and no additional effects of environment or geographic distance could be detected.

Attention has recently been given to the influence of local adaptation on genetic patterns via the ‘general barriers’ mechanism (Nosil et al. 46), and several studies have found evidence for a role of environmental selection in shaping neutral marker patterns (Nosil et al. 46; Freeland et al. 20). In the light of the strong environmental gradient in the present study area (Tables 1 and S1) there should be ample opportunities for local adaptation in populations of *E. sativa*. However, although the existence of IBA (isolation by adaptation) patterns seems relatively common (Nosil et al. 46) we could not detect a distinct influence of the environment on genetic diversity. It is certainly possible that neither geography nor environment plays a significant role in this system or that selection is too weak to produce local adaptation, although intuitively this seems unlikely. Accordingly, explanations could be that the scale at which most of the habitat differentiation is found is greater than that at which gene flow occurs, which would lead to neutral patterns uncorrelated with the environment; or, alternatively, there could be selection pressures unrelated to the environmental axes used here. On the other hand, the presence of a relatively high correlation among the three explanatory variables (environment, geography, and cluster) might mask any smaller effects of geography or environment (Mulley et al. 39). At any rate, an accurate estimation of relative effects does not seem possible when variables are correlated. Indeed, this may be common in nature; in many cases the effects of genetic barriers or geographic distance, on one hand, and adaptation to local habitat conditions, on the other hand, may not be independent because the homogenizing effects on gene flow counteracts differentiation and local adaptation (Slatkin 58). Thus, some form of spatial restriction of gene flow between populations (e.g., distance, geographical barriers) might often be necessary for local adaptation to occur.

Phenotypic variation is thought to be more readily affected by selection than molecular variation, as the former may be the direct target of selection. Indeed, significant correlation of the phenotype data from the present common-garden experiment with the environmental characteristics implies a possible role for selection. In this case, the finding that edaphic factors accounted for more variation than climatic factors disentangles it somewhat from the geographic component. It is, however, difficult to speculate about which traits are influenced by the soil type. Indeed, most plant features monitored in the present study (i.e., performance-related characters, trichome density, and herbivore damage), as well as observed variation in seed germination in response to photo-thermal cues (Barazani et al. 6), can reasonably be attributed to climatic factors. Similarly, phenotype variations in East-Mediterranean plant populations have largely been associated with climatic conditions, encompassing phenology, morphology, and ecophysiological differences such as seed dormancy (Gutterman 22; Yonash et al. 101; Yan et al. 72). The contrasting lack of significant regression coefficients with the data from the insect-free net-house experiment may seem surprising at first glance as several traits were measured in both experiments. The most likely explanation is that these differences in traits resulted from the response of the plants to the ambient conditions in the common garden experiment – for example, herbivory, radiation, etc.

### Detection of outlier loci

The three programs, Dfdist, Bayescan, and SAM detected 1.6, 2.2, and 3.2%, respectively, of all polymorphic loci as outliers; the total proportion of outliers was 5.4% (Table 4). These numbers are within the range found in other plant species, and are slightly below the average proportion of outliers (8.5%) found in other studies (Strasburg et al. 60). However, findings like these are difficult to compare, because the studies described by example, Strasburg et al. (60) and Nosil et al. (46) differ in the methods used and the significance levels chosen, as well as in their study systems. In *E. sativa*, in the present study, only three of the nine candidate loci (55GA170, 55GA254, 54GA254) were identified by more than one method. However, in light of the fact that the programs use different methods to detect candidate loci, it is not surprising that the different programs identified different markers as outliers. Even when only comparing differentiation based outlier methods, several papers have shown that programs perform differently using both experimental and simulated data, for example, (Vitalis et al. 65; Foll and Gaggiotti 19; Excoffier et al. 18; Nunes et al. 47).

Several researchers have raised concerns about the possibilities of high rates of false positives in genome scans for outliers (Foll and Gaggiotti 19; Excoffier et al. 18). The finding of a hierarchical genetic structure, for example, may have an influence on the detection of outliers. Excoffier et al. (18) found a large number of false positives when using an island model of differentiation in a hierarchical population structure. In their scenarios, simulations showed that the rate of false positives for loci under divergent selection was high when differentiation between groups was strong, compared to among population differentiation, but relatively low when the structure among groups was less pronounced. In our present study, we included all populations in a global analysis, to ensure that the data would cover as much of the climatic gradient as possible.

However, the pattern of differentiation in *E. sativa* appears to correspond more to the case described by Excoffier et al. (18) which produced fewer false positives; if we exclude outliers from our data set the amount of among-group and within-group variation in AMOVA changes to 6% and 5%, respectively. We therefore assume that the relatively strict *P*- and *q*-value thresholds applied prevent a problematic rate of false positives. Nevertheless, we further tested whether pooling populations within the two clusters (excluding the admixed MZ) affected the detection of outliers with Dfdist. In this analysis fragments 55GA170 and 54GA254 were again identified, whereas fragment 55GA254 was not (Fig. S5). However, fragment 55GA254 showed a pattern of differentiation that was most marked among populations within the genetic groups, therefore its detection should not be affected by the hierarchical structure. This was also true of the two additional outlier loci detected by Bayescan, which suggests to us that the results of the outlier analyses are robust with respect to hierarchical structure.

Slightly more of the candidate loci detected by SAM were associated with the first PCA axis, which related mainly to temperature and rainfall, than with the second, which related mainly to LOI, CaCO_3,_ and SSA, but also to topography. If we include candidate loci with nonsignificant associations, seven out of nine loci were most strongly associated with PC1. However, this is not significantly more than for PC2 (sign test, *P *=* *0.18); furthermore, it may reflect the correlation between subclusters and climate, which might influence the results of this analysis. Thus, candidate loci in *E. sativa* showed no clear trend toward closer association with climatic than with other environmental factors. Most studies that test for associations between marker and environmental data use climatic data, most likely because of their availability. However, our results suggest that incorporating other data such as edaphic conditions might be a promising strategy; and this is not surprising in the light of the importance of edaphic factors in ecotypic variation (e.g. Turesson 62; Heywood 28; Sambatti and Rice 55).

Of the five *F*_ST_ outlier loci found in the present study, only two showed significant associations with the environment (Table 4). The three remaining outliers showed distributions of allele frequencies that did not correspond to any of the considered patterns – that is, IBD, IBA, PCs – and also did not show the north-south pattern of genetic substructure (data not shown). This lends further support to the idea that additional environmental parameters should be identified to help explain the neutral patterns of genetic differentiation. However, even if we were confident in the significant associations found, it would still be difficult to make predictions about the selective forces involved, as well as their targets. For example, any locus associated with rainfall is likely to be associated with temperature also, because of their high correlation, and further experiments would be needed to confirm whether one, both, or neither of these factors acted as selective agent(s). In the present case, however, the available phenotype data might help to provide further insights into these questions. Thus, we correlated the phenotype means with band frequencies of outlier loci in order to associate candidate loci with phenotypic traits. After correcting for multiple comparisons, and removing trait combinations with high correlations (*r* > 0.7), we found two correlations that were significant for fragment 54GA254, the only candidate identified by all three methods: (1) stem trichome density (net-house experiment, Pearson's *r* = −0.95, *P *=* *0.033); and (2) late herbivore damage (common-garden experiment, *r* = 0.96, *P *=* *0.010). It is well known that trichome production is induced under herbivore attack (Yoshida et al. 73; Sletvold et al. 59), which indicates a clear relationship between trichome density and plant defense against herbivore damage. In addition, it can be assumed that in the common-garden experiment the trichome density resulted from exposure to herbivores in the field, which might explain why fragment 54GA254 was correlated with trichome density in the insect-free net-house and not in the common-garden experiment. Furthermore, in many plants trichomes reflect excess light and dissipate heat (Vogelmann 66), and thus might also have a direct association with climate, as indicated by the present SAM analysis. Thus, there is a distinct possibility that this marker is linked to a locus that regulates trichome development and, therefore, that could have different adaptive value across the range of this study. Isolation and sequencing of this and the other candidate loci are still needed, in order to identify linkages to known genes and, therefore, possible roles in local adaptation.

There is no doubt that more experiments are needed to determine the local selective pressures and the underlying relationships between genotype, phenotype, and the environment. The use of new approaches that utilize phenotypic data from genotyped individuals within populations in exploratory genome scans (Herrera and Bazaga 27; Herrera 26) would have enabled us to infer associations between traits and candidate loci at the individual level, and to directly estimate the genetic component of the phenotypic variation. Nevertheless, the approaches taken in the present study enabled us to formulate hypotheses about the selective pressures experienced by the various populations along an environmental gradient, and to speculate about the targets of selection – thereby demonstrating the potential value of combining environmental and phenotypic data with detailed genetic surveys.

## Supporting Information

**Figure S1.** Assessment of flower color, herbivore damage, and trichome density were based on qualitative evaluation of these traits as are illustrated in the pictures: (A) petal color ranging from pale cream (left) to yellow (right); (B) herbivore damage, in a scale of low damaged plants (left) to damaged plants (right); (C) low and high trichome density on stems and flower buds.

**Figure S2.** Results of the principal component analysis (PCA) of the site of *E. sativa* populations and environmental climatic and edaphic characteristics (see Tables  and S1).

**Figure S3.** Results of the principal component analysis (PCA) of the phenotypic data from (A) the net-house experiment and (B) the common-garden experiment. The first two axes account for 72% and 65%, respectively, of the variation in (A) and (B).

**Figure S4.** A UPGMA dendrogram of *E. sativa* populations, based on Nei genetic distances of the AFLP data.

**Appendix S1.** Phenotypic evaluation (mean values) in populations of *E. sativa* obtained in the common-garden and net-house experiments, and plant height measured in their natural habitats.
