# Supplementary figures and images for: Environmental effects on molecular and phenotypic variation in populations of Eruca sativa across a steep climatic gradient
Source: Ecol Evol. 2013 Jun 24;3(8):2471–84. doi: 10.1002/ece3.646 (PMC3930051; doi:10.1002/ece3.646)

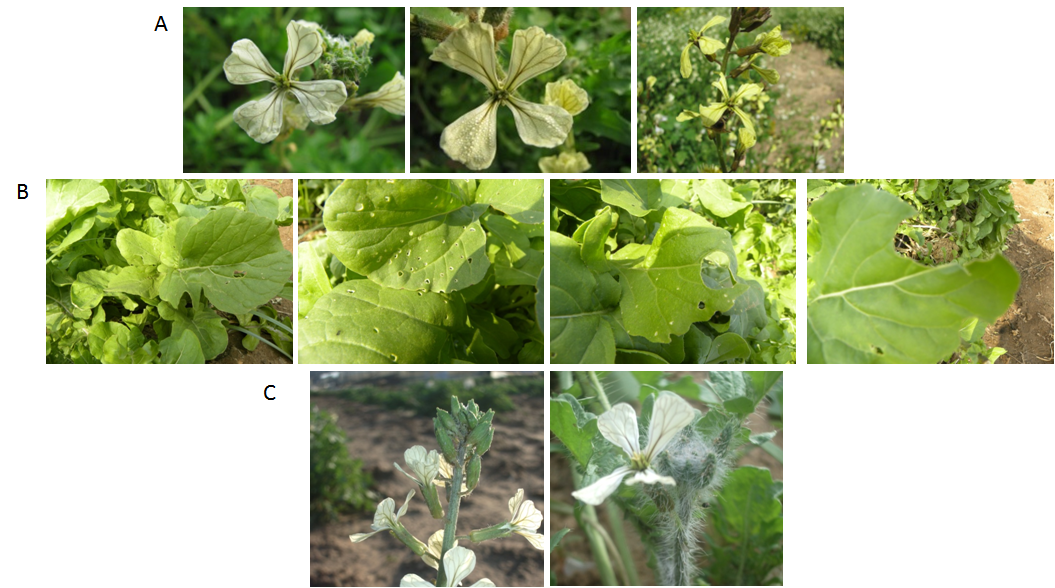

Supplement: Supplementary file 1 — Figure S1. Assessment of flower color, herbivore damage, and trichome density were based on qualitative evaluation of these traits as are illustrated in the pictures: (A) petal color ranging from pale cream (left) to yellow (right); (B) herbivore damage, in a scale of low damaged plants (left) to damaged plants (right); (C) low and high trichome density on stems and flower buds. [file ece30003-2471-SD1.tif]

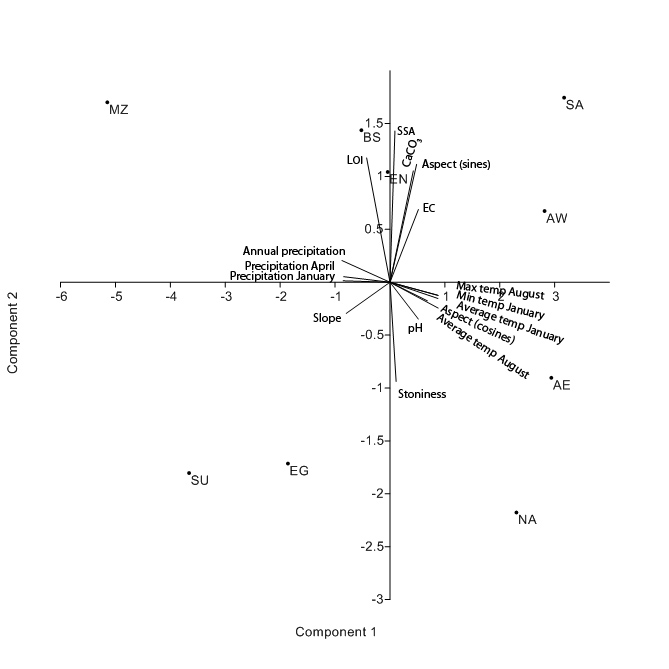

Supplement: Supplementary file 2 — Figure S2. Results of the principal component analysis (PCA) of the site of E. sativa populations and environmental climatic and edaphic characteristics (see Tables and S1). [file ece30003-2471-SD2.tif]

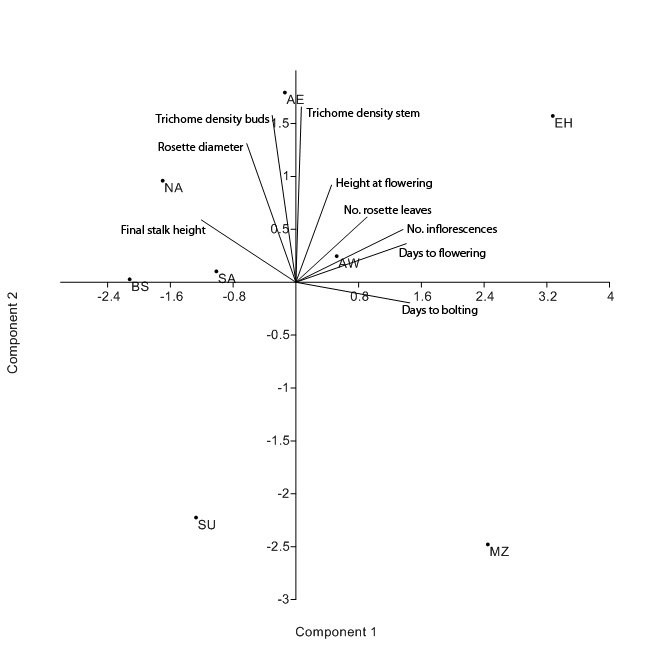

Supplement: Supplementary file 3 — Figure S3. Results of the principal component analysis (PCA) of the phenotypic data from (A) the net-house experiment and (B) the common-garden experiment. The first two axes account for 72% and 65%, respectively, of the variation in (A) and (B). [file ece30003-2471-SD3.tif]

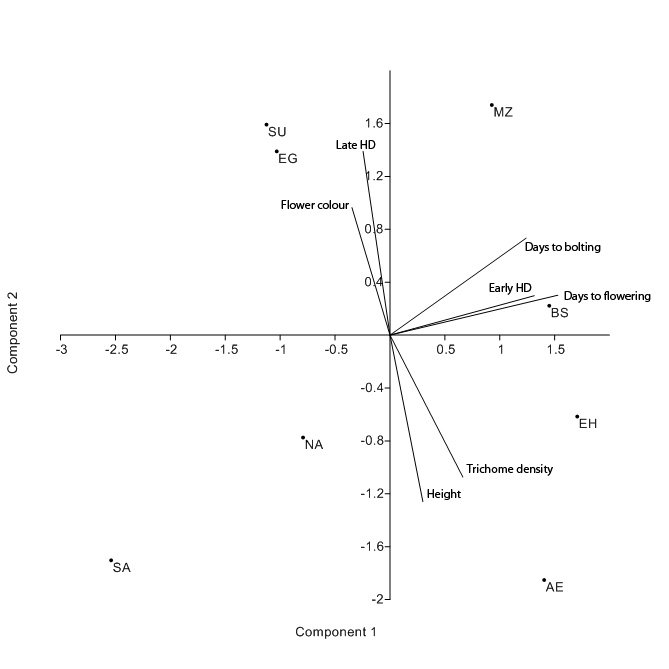

Supplement: Supplementary file 4 [file ece30003-2471-SD4.tif]

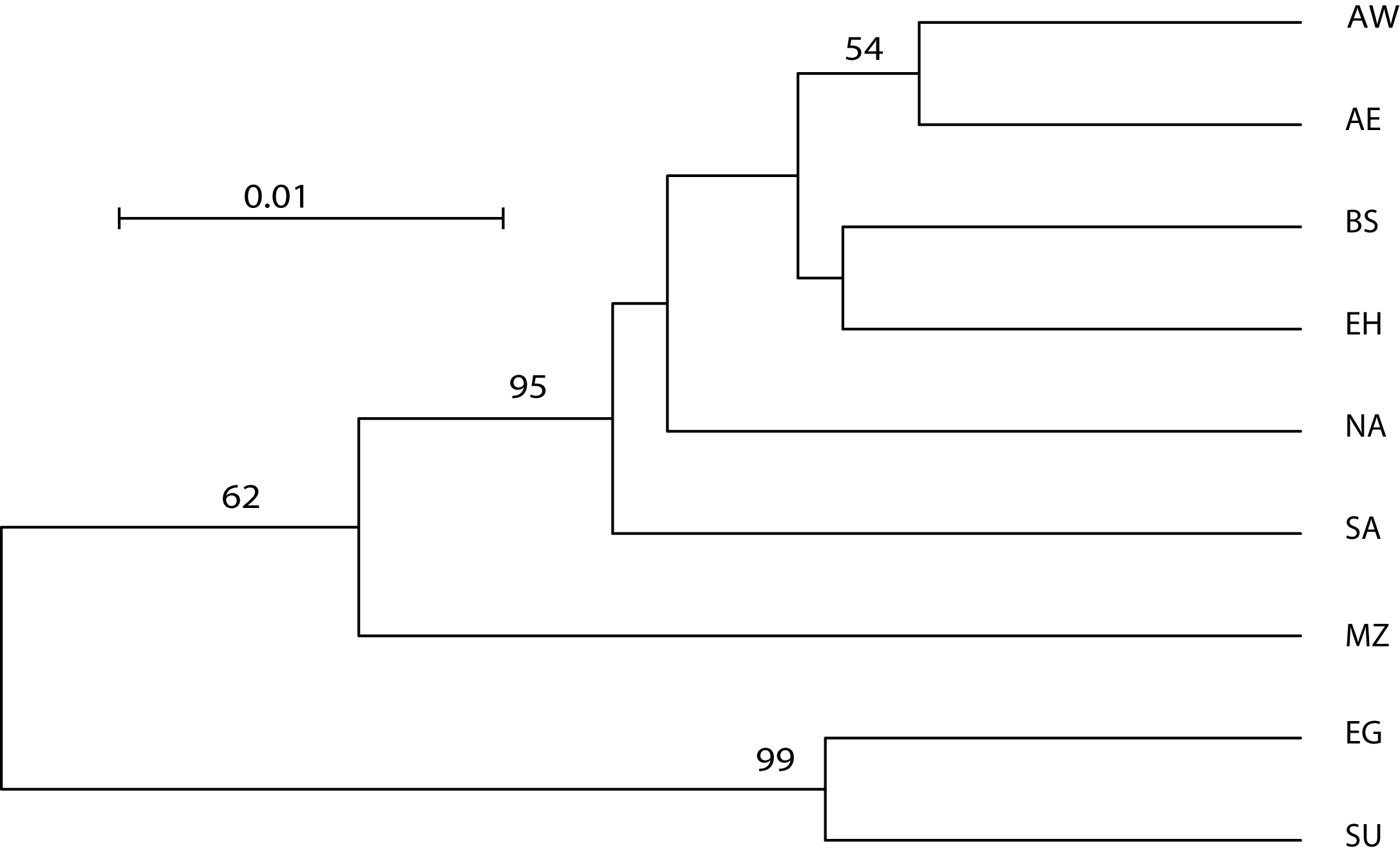

Supplement: Supplementary file 5 — Figure S4. A UPGMA dendrogram of E. sativa populations, based on Nei genetic distances of the AFLP data. [file ece30003-2471-SD5.tif]

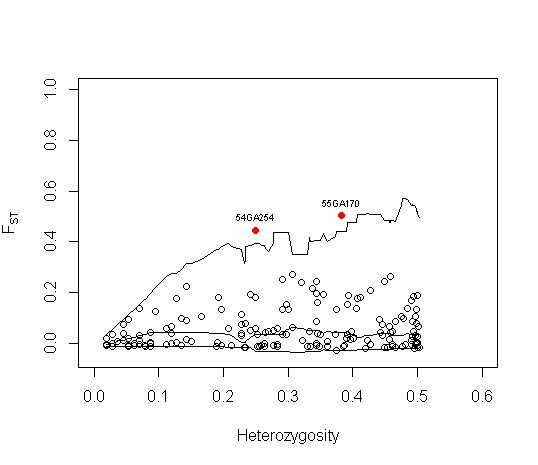

Supplement: Supplementary file 6 [file ece30003-2471-SD6.tif]
