# Supplementary material for: Environmental effects on molecular and phenotypic variation in populations of Eruca sativa across a steep climatic gradient
Source: Ecol Evol. 2013 Jun 24;3(8):2471–84. doi: 10.1002/ece3.646 (PMC3930051; doi:10.1002/ece3.646)
Supplement: Supplementary file 7 [file ece30003-2471-SD7.docx]

**Table S1.**

|  | **Aspect^*^** | **Slope (°)** | **Average values of edaphic characteristics** | | | | | |  |
| --- | --- | --- | --- | --- | --- | --- | --- | --- | --- |
|  |  |  | **Stoniness^**^** | **SSA**  **(m^2^ g^-1^)** | **CaCO_3_**  **(%)** | **LOI (%)** | **pH** | **EC**  **(mS)** | |
| SU | 217.9 | 18.9 | 0.7 | 53 | 40 | 2.09 | 8.3 | 0.22 | |
| EG | 279.7 | 8.2 | 0.2 | 65 | 17 | 1.98 | 8.2 | 0.16 | |
| MZ | 138.3 | 16.0 | 0.6 | 131 | 62 | 4.98 | 8.0 | 0.31 | |
| BS | 75.1 | 3.1 | 0.5 | 114 | 69 | 5.59 | 8.1 | 0.30 | |
| EH | 67.2 | 3.4 | 0.3 | 79 | 87 | 4.37 | 8.1 | 0.32 | |
| AW | 66.0 | 2.8 | 0.2 | 155 | 62 | 1.39 | 8.4 | 2.36 | |
| AE | 86.1 | 1.2 | 1.0 | 51 | 80 | 1.57 | 8.4 | 0.54 | |
| SA | 82.2 | 2.2 | 0.2 | 174 | 65 | 2.78 | 8.3 | 5.24 | |
| NA | 33.9 | 3.0 | 1.6 | 20 | 53 | 1.62 | 8.1 | 0.87 | |

^*^Relative to the north (0°); ^**^The ratio between soil fractions bigger and smaller than 2 mm. For population abbreviations see Table 1.
